# Irritability in boys with autism spectrum disorders: an investigation of physiological reactivity

**DOI:** 10.1111/jcpp.12382

**Published:** 2015-01-28

**Authors:** Nina Mikita, Matthew J. Hollocks, Andrew S. Papadopoulos, Alexandra Aslani, Simon Harrison, Ellen Leibenluft, Emily Simonoff, Argyris Stringaris

**Affiliations:** ^1^ Department of Child and Adolescent PsychiatryInstitute of Psychiatry, Psychology & NeuroscienceKing's College LondonLondonUK; ^2^ Department of Psychological MedicineInstitute of Psychiatry, Psychology & NeuroscienceKing's College LondonLondonUK; ^3^ Section on Bipolar Spectrum DisordersNational Institute of Mental HealthBethesdaMDUSA

**Keywords:** Autism spectrum disorders, irritability, cortisol, heart rate, psychosocial stress test

## Abstract

**Background:**

Irritability in people with autism spectrum disorders (ASD) is common and impairing, yet its mechanisms remain understudied. We investigated symptom reporting and mechanisms of irritability in ASD, focusing on the relation between irritability and physiological stress responses.

**Methods:**

Forty‐seven unmedicated boys with high‐functioning ASD (hfASD) and 23 typically developing boys aged 10–16 years completed a psychosocial stress test. Changes in cortisol, heart rate and heart rate variability throughout the test were recorded. Self‐ and parent‐reported measures of irritability were obtained. Irritability symptom reporting in the hfASD group was compared to two groups of boys without ASD: highly irritable boys (severe mood dysregulation, SMD;* n *=* *40) and healthy‐control boys (HC;* n* = 30).

**Results:**

Boys with hfASD scored significantly higher on irritability than HC boys, and they reported a pattern of irritability symptoms closely resembling that of boys with SMD. The internal consistency of irritability in hfASD was high by parent‐ and self‐report. Although boys with hfASD showed significant stress‐induced changes in cortisol and heart rate, those who rated themselves as highly irritable had lower cortisol levels throughout the test compared to those low on irritability. Participants rated as highly irritable by their parents showed blunted cortisol and heart rate responses to stress. The effects of irritability on heart rate, but not cortisol, were accounted for by trait anxiety.

**Conclusions:**

Irritability can be measured reliably in hfASD and is associated with distinct biological responses to stress.

## Introduction

Autism spectrum disorders (ASD) are characterized by deficits in social reciprocity and communication, and by restricted, repetitive behaviors (APA, 2013). Additionally, children with ASD often display high levels of irritability (Mandy, Roughan, & Skuse, 2014; Simonoff et al., 2012). However, little research has been conducted on the mechanisms of irritability in children with ASD. This is surprising because irritability in typically developing (TD) children is associated with long‐term adverse outcomes (Leibenluft, 2011; Mikita & Stringaris, 2013). Here, we use a multimethod, multi‐informant experimental approach to investigate irritability in boys with high‐functioning ASD (hfASD).

In previous studies of children with ASD, the term ‘irritability’ was often used to describe severe behavioral difficulties, e.g., verbal and physical aggression, self‐injury or property destruction. Such behaviors feature, for example, on the irritability subscale of the Aberrant Behavior Checklist (ABC; Aman, Singh, Stewart, & Field, 1985). By contrast, in TD children, irritability refers to a mood that may or may not lead to aggression (Leibenluft, 2011; Mikita & Stringaris, 2013; Stringaris, Goodman, et al., 2012). Despite these purported differences, recent studies suggest that irritability in ASD and TD youth may share important characteristics. For instance, irritability in TD children has stronger phenotypic and genetic associations with depression than with delinquency (Stringaris, Zavos, Leibenluft, Maughan, & Eley, 2012). Likewise, in a cross‐sectional study of children with ASD, Mandy et al. (2014) identified that while DSM‐5‐defined argumentative and defiant behavior was associated with externalizing problems, angry/irritable symptoms predicted internalizing problems. It is important to distinguish between irritable mood and acts of hostility or aggression, as their mechanisms may be different (Stringaris, 2011). An additional problem with scales such as the ABC is that they were originally developed for people with intellectual disability and include symptoms, such as screaming, that are less common in hfASD (Arnold et al., 2003). Moreover, there is a need for scales that are not just observer‐ or parent‐rated but also incorporate the views of people with ASD themselves.

In TD children, chronic irritability was studied extensively under the term severe mood dysregulation (SMD), characterized by frequent temper outbursts with irritability between outbursts (Leibenluft, Charney, Towbin, Bhangoo, & Pine, 2003). Childhood SMD predicts depression in adolescence (Brotman et al., 2006), consistent with the well‐established longitudinal association between irritability and internalizing problems (Krieger et al., 2013; Mandy et al., 2014; Stringaris & Goodman, 2009a, 2009b). Recently, Simonoff et al. (2012) demonstrated that parent‐reported mood dysregulation symptoms identified adolescents with ASD who had higher rates of comorbidity. This suggested that mood dysregulation in young people with ASD might resemble that of TD children. However, mood dysregulation was not limited to irritability but broadly defined using questions about sad mood, mood lability and explosive rage and did not include data on self‐reported irritability.

Our study addresses the gaps in existing literature by focusing on two sets of questions. The first concerns recognition and measurement of irritability in young people with hfASD. Arguably, children with hfASD may underreport their irritability symptoms due to introspection difficulties, as proposed by Mazefsky, Kao, and Oswald (2011) who found low correspondence between a parental diagnostic interview and self‐reports of depression, anxiety and ADHD in youth with hfASD. Here, we measure irritability using the self‐ and parent‐reported Affective Reactivity Index (ARI; Stringaris, Goodman, et al., 2012), which was previously shown to be reliable in TD children (DeSousa et al., 2013; Mulraney, Melvin, & Tonge, 2014; Stringaris, Goodman, et al., 2012) and distinguished between children with SMD and healthy controls (Stringaris, Goodman, et al., 2012). We assess consistency of reporting and compare the symptom pattern of irritability in boys with hfASD to that of boys with severe irritability (SMD) and healthy controls. We also investigate whether the strong cross‐informant agreement for irritability symptoms in TD children (Stringaris, Goodman, et al., 2012) is present in our hfASD sample.

The second set of questions concerns mechanisms underlying irritability in ASD. We test the hypothesis that irritability may be associated with how individuals with ASD respond to stress. Physiological mechanisms of stress‐response include the autonomic nervous system (ANS) and the hypothalamic‐pituitary‐adrenal (HPA) axis. Under threat, activation of the sympathetic branch of the ANS prepares an individual to deal with the stressor, resulting in heightened arousal, e.g., increased heart rate (HR). The body's return back to homeostasis is controlled by the parasympathetic branch of the ANS. The HPA axis, which encompasses a cascade of biochemical reactions, is also activated following stress exposure, resulting in increased levels of cortisol that peak around 20 min post stressor (Romanczyk & Gillis, 2006). In TD adults, Moons, Eisenberger, and Taylor (2010) distinguished between self‐reported anger and fear responses to stress, which differentially influenced the HPA axis. Anger‐driven, confrontational stress responses were associated with greater stress‐induced increase in cortisol, while fear reactions were associated with a decrease in cortisol levels. This suggests that a tendency to respond to stress in an irritable manner may be associated with a distinct pattern of physiological activation. However, self‐reported anger and fear were moderately correlated in the Moons et al. (2010) study, making disentanglement of the two emotions difficult. Studies that induced stress experimentally in children with ASD, using psychosocial stress paradigms such as the Trier Social Stress Test (TSST; Kirschbaum, Pirke, & Hellhammer, 1993), have been inconsistent. While most studies found a blunted cortisol response to psychosocial stressors (Lanni, Schupp, Simon, & Corbett, 2012; Levine et al., 2012), these results were not always replicated (Jansen, Gispen‐de Wied, van der Gaag, & van Engeland, 2003). Research into peripheral physiological responses to stress, e.g., HR changes, also produced mixed results. While some studies found children with ASD to physiologically respond differently to stressors relative to controls (Goodwin et al., 2006), others found no such differences in HR stress‐responsiveness (Jansen et al., 2003; see Appendix S1 for a fuller list).

This inconsistency may be partly explained by the relative contributions of irritability and anxiety in stress‐response. Research suggests that youth with ASD show greater levels of anxiety than those in community populations, and that anxiety levels of children with ASD are comparable to those of clinically anxious children (for a review see MacNeil, Lopes, & Minnes, 2009). Furthermore, mood dysregulation is more common in adolescents with ASD who display symptoms of anxiety (Simonoff et al., 2012) and it was suggested that irritability may worsen when a person with ASD becomes anxious (Tantam, 2003). Additionally, irritability is common and impairing among TD children with anxiety disorders (Krebs et al., 2013; Stoddard et al., 2014). It is therefore important to examine how irritability is related to stress responses in children with ASD and how this relation is influenced by co‐occurring anxiety. As mentioned above, the two emotions may be difficult to distinguish.

We use a multimethod, multi‐informant experimental approach to investigate irritability, anxiety, and physiological reactivity to stress in boys with hfASD. We first investigate whether irritability can be measured reliably in boys with hfASD using a concise scale. Secondly, we examine irritability and anxiety in boys with hfASD in relation to their HR, HR variability, and cortisol levels following a psychosocial stress test.

## Methods

### Sample

#### Participants with hfASD

Fifty‐four male participants with hfASD aged 10–16 (see Table 1) were recruited from clinics in London and the south‐east of the United Kingdom. All participants had a full‐scale IQ≥70 on the Wechsler Abbreviated Scale of Intelligence (WASI; Wechsler, 1999) and were not taking psychotropic medications. ASD diagnoses were made by expert clinicians and, in 31/54 cases, confirmed using either the Autism Diagnostic Interview‐Revised (ADI‐R, Lord, Rutter, & Couteur, 1994) or the Autism Diagnostic Observation Schedule‐Generic (ADOS‐G, Lord et al., 2000). In the absence of ADI/ADOS confirmed diagnosis, a Social Communication Questionnaire (SCQ; Rutter, Bailey, Lord, & Berument, 2003) score of ≥15 and clinical diagnosis were required. Two participants were excluded based on this criterion. Of the remaining sample, we obtained irritability measurements from 47 participants.

**Table 1:** Means (standard deviations, ranges) and sample sizes for key variables in the study in participants with high‐functioning autism spectrum disorders (hfASD) and typically developing (TD) controls

	hfASD	*n*	TD controls	*n*
Participant characteristics				
Age	12.8* (2.0,10–16)	52	13.9* (1.9, 10–16)	23
IQ	101.2*** (13.5, 76–138)	52	117.7*** (9.1, 96–136)	23
SCQ	23.2*** (6.4, 12–36)	50	1.5*** (1.5, 0–6)	23
Irritability				
Parent‐reported	7.6*** (3.0, 0–12)	44	0.6*** (0.8, 0–3)	20
Self‐reported	5.1* (3.1, 0–12)	29	2.6* (1.7, 0–5)	9
Anxiety				
Parent‐reported	33.9*** (19.0, 3–88)	50	6.9*** (5.1, 0–24)	23
Self‐reported	31.1*** (15.8, 3–72)	50	12.0*** (6.4, 1–25)	22
Psychosocial stress test				
Subjective stress rating				
Before test	2.0 (1.9, 1–9)	46	1.6 (1.2, 1–6)	23
After test	5.2 (2.6, 1–10)	43	4.6 (2.2, 1–8)	22
*log* cortisol				
Before test	1.4 (0.4, 0.6–2.4)	52	1.4 (0.3, 0.7–2.0)	23
After test	1.5** (0.4, 0.5–2.2)	50	1.8** (0.5, 1.0–3.1)	22
Heart rate (bpm)				
Before test	84.4** (11.1, 62.9–109.8)	51	76.5** (9.2, 60.2–93.6)	23
During test	89.3 (11.2, 67.1–120.8)	50	87.0 (10.8, 66.6–103.9)	22
After test	80.9** (11.2, 57.4–105.3)	49	72.9** (9.1, 59.1–90.5)	20

*p < .05; **p < .01; ***p < .001.

#### Non‐ASD participants

Two independent control samples of boys without ASD were used, each to answer a different research question. First, to answer the question about measurement of irritability in ASD, we used 40 boys with SMD (age 12.6 ± 2.6, 8–17 years) and 30 healthy‐control (HC) boys (age 11.5 ± 3.6, 6–18 years) studied in our previous published work on youth irritability (Stringaris, Goodman, et al., 2012). This sample provided data on self‐ and parent‐reported irritability (ARI), but did not complete the stress test. Second, to examine the role of irritability in shaping physiological responses to stress, we used an independent sample of 23 TD boys aged 10–16 who completed both the psychosocial stress test and ARI questionnaires. This sample was recruited from local London schools and through public advertisement, concurrently with our ASD sample, and had no parent‐reported history of psychiatric or neurological problems.

The study of physiological stress responses was approved by the South East London Research Ethics Committee (10/H0870/67). Written informed consent was obtained from all participants.

### Symptom assessment

*Irritability* was measured using the Affective Reactivity Index (ARI; Stringaris, Goodman, et al., 2012), a 6‐item scale that is both parent‐ and self‐reported. The ARI asks about symptoms of irritability in the previous 6 months and includes a 7th item assessing impairment due to irritability. The scale showed excellent internal consistencies in TD children, with Cronbach's alphas 0.89 (parent‐report) and 0.90 (self‐report; Stringaris, Goodman, et al., 2012).

*SMD* was assessed using a supplementary module of the Schedule for Affective Disorders and Schizophrenia (K‐SADS‐PL; Kaufman et al., 1997; Leibenluft et al., 2003) and defined by persistent abnormal mood (anger or sadness), hyperarousal and increased reactivity to negative emotional stimuli (for full SMD criteria see Leibenluft et al., 2003). SMD precludes the diagnosis of ASD or bipolar disorder.

*Trait anxiety* was measured with the Spence Child Anxiety Scale (SCAS; Spence, 1998) parent and child versions, a 44‐item screening questionnaire providing global measurement of most childhood anxiety symptoms.

### Procedure

*The Psychosocial Stress Test* (*PST)* was carried out in the afternoon, beginning between 1300 h and 1400 h, to reduce the impact of diurnal cortisol variation. Participants were asked not to consume any food or drink within 30‐min of task initiation. Participants were told they would undertake a mildly stressful task, preceded by 40 min of relaxation and followed by a 40‐min recovery period in which they would watch cartoons. The BioHarness HR telemetry system was then placed onto the participants.

The PST was a modified version of the TSST (Kirschbaum et al., 1993) with the mental arithmetic task replaced with the Rey‐Osterrieth Complex Figure test (Osterrieth, 1944; Rey, 1941). During the 20‐min stress paradigm, participants were asked to: copy a complex figure, prepare a speech about themselves in 10 min, give a 5‐min presentation, and remember and reproduce the complex figure under timed conditions. Up until the end of speech preparation, two researchers were present in the room. A third person (unknown to the participant, presented as an evaluator) then entered the room asking the participant to begin their presentation. Participants were required to stand for 5 min and standardized prompts were given after 30s of silence.

*Salivary cortisol* was collected six times throughout the PST: twice during rest (−40 min, −20 min), prestressor (0 min), post stressor (+20 min) and twice during recovery (+40 min, +60 min). Saliva samples were collected in plain Sarstedt salivettes and stored at −40°C. Saliva cortisol concentrations were determined using ‘Immulite’ Siemens Immunoassay system (www.diagnostics.siemens.com; Mondelli et al., 2010).

*Heart Rate (HR)* was recorded continuously throughout the PST. HR electrocardiogram (ECG) was measured at 250 Hz using the Zephyr BioHarness wireless telemetry system.

The ECG signal was recorded and analyzed with Labchart 7 (ADInstruments Pty Ltd, Bella Vista, Australia). Mean HR values were extracted and divided into five segments: first 20 min of rest; second 20 min of rest; 20 min of stress test; first 20 min of recovery and second 20 min of recovery, in order to mirror cortisol analyses. HR variability (HRV) analysis was conducted using the Labchart HRV module (see Appendix S2 for details).

*Subjective stress* responses (rated on a scale from 1 to 10) were collected six times during the PST following salivary cortisol collection.

### Data analysis

We analyzed data with SPSS 20 (IBM Corp, 2011) and Stata 11 (StataCorp, 2009). Cortisol was nonnormally distributed and therefore was *l*og‐transformed. We collected a total of 44 parent‐reported and 29 self‐reported ARI questionnaires for participants with hfASD (26 had both self‐ and parent‐reported ARI, three only had self‐ and 18 only had parent‐reported ARI). For TD controls, we collected 20 parent‐reported and nine self‐reported questionnaires (11 had ARI data from both informants). There were no differences in IQ between participants with hfASD who completed the ARI and those who did not. Missing data for other key variables were limited (see Table 1). Age effects on all variables were examined using one‐way analyses of variance (ANOVAs) with age as a continuous variable. Age had an effect on cortisol levels during the prestress rest period in the hfASD group (*p *=* *.002) and was therefore added as a covariate in hfASD cortisol analyses.

#### Measurement of irritability in hfASD

Cronbach's alpha coefficients assessed internal consistency of the ARI in boys with hfASD versus controls. Pearson correlation coefficients estimated the parent‐child reporting correspondence. We then compared the item distribution pattern of the ARI in boys with hfASD to that of boys with SMD and HC boys. Finally, we examined how the severity of irritability symptoms related to impairment using ANOVA on ARI total score and ARI impairment item.

#### Irritability and anxiety

Pearson correlation coefficient estimated the relation between irritability and SCAS scores, separately for self‐ and parent‐report.

#### Psychosocial stress test

Paired‐samples t‐tests and repeated‐measures ANOVAs were performed to examine the efficacy of the PST in producing stress‐induced changes to cortisol and HR, separately for hfASD and TD controls.

#### Irritability and physiological responses to stress in hfASD

Consistent with previous studies (van Goozen et al., 1998; Lanni et al., 2012) we conducted repeated‐measures ANOVAs, with continuous irritability score entered as a covariate, separately for self‐ and parent‐report. The effects of anxiety on physiological stress‐responsiveness were assessed by adding trait anxiety (parent‐ or self‐reported SCAS) as a covariate. For illustration purposes, we used a median split (low vs. high ARI score). As we were primarily interested in physiological reactivity to the experimental stressor, only the time points that directly test our hypothesis were used in the analysis. For cortisol, these were immediately before (0 min) and immediately after the stressor (+20 min). For HR, three middle phases were used: second rest phase served as a baseline and was compared to the stress condition, while the first recovery period was used to evaluate return to baseline post stressor. ANOVA analyses were complemented by piecewise linear regression models, which are reported in Appendix S3.

## Results

### Reliability and item profile of irritability in boys with hfASD

#### Internal consistency

Our first aim was to check whether the irritability scales were internally reliable in hfASD. The ARI showed excellent internal consistency with Cronbach's alphas 0.82 (parent‐report) and 0.80 (self‐report). This was compared to 0.91/0.86 in boys with SMD, 0.83/0.21 in HC boys (note: 15 self‐reports) and 0.43/0.31 in TD boys (note: nine self‐reports).

#### Cross‐informant agreement

Next, we checked whether irritability reported by boys with hfASD related to their parents’ report of these problems. There was a moderately high correlation between parent‐ and self‐report scales, *r*(26) = .55, *p *=* *.003. Irritability scores were significantly higher for parent‐ than self‐report in boys with hfASD (7.50 ± 2.66 vs. 5.35 ± 2.98; *t*(25) = 4.10, *p *<* *.001, *d *=* *0.76). Similar differences between parent‐ and self‐reported irritability were observed in boys with SMD (6.98 ± 3.25 vs. 4.70 ± 2.78, respectively) and HC boys (1.34 ± 2.00 vs. 0.27 ± 0.46, respectively). Cross‐informant coefficients for SMD, HC and TD groups were 0.60, 0.43, and 0.64, respectively.

#### Item distribution

**Figure 1:**
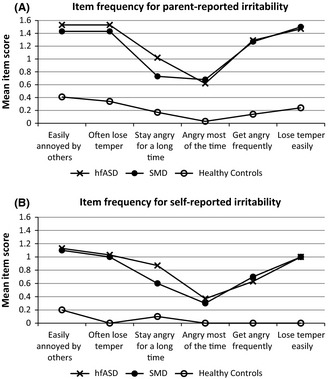
Item frequencies for parent‐ and self‐reported irritability in boys with high‐functioning autism spectrum disorders (hfASD) compared to boys with severe mood dysregulation (SMD) and healthy controls

We then compared the symptom pattern of irritability in boys with hfASD to that of boys with SMD and HC boys. The pattern of irritability symptoms in hfASD closely matched to SMD (Figures 1A and 1B). Being easily annoyed was the most common item, while the duration item ‘angry most of the time’ was reported least by both reporting sources. HC boys scored significantly lower than those with SMD or hfASD on all items of the ARI.

#### Impairment

**Figure 2:**
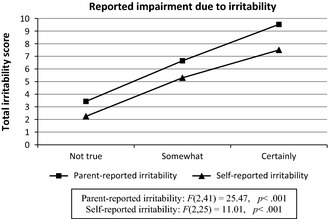
The relation between impairment due to irritability and total irritability score in boys with high‐functioning autism spectrum disorders (hfASD)

Our next aim was to investigate the extent to which boys with hfASD perceive their irritability as impairing. Indeed, increases in irritability symptoms were strongly associated with increases in reported impairment due to irritability, by either reporting source (Figure 2).

### Irritability and anxiety

Parent‐reports of irritability and anxiety were strongly correlated, *r*(44) = .49, *p *=* *.001. In contrast, self‐reported irritability did not correlate significantly with self‐reported anxiety, *r*(28) = .052, *p *=* *.791. No significant correlations between irritability and anxiety were found in TD boys; *r*(20) = .219, *p *=* *.353 (parent‐report) and *r*(9) = .400, *p *=* *.286 (self‐report).

### Psychosocial stress test

There was a significant and statistically equal [*F*(1,63) = 1.32, *p *=* *.256] rise in subjective stress for boys with hfASD [*t*(42) = 8.13, *p *<* *.001, *d *=* *1.37] and TD boys [*t*(21) = 6.14, *p *<* *.001, *d *=* *1.60]. The psychosocial stressor significantly increased cortisol levels in boys with hfASD, *t*(49) = 2.10, *p *=* *.041, *d *=* *0.30, and TD boys, *t*(21) = 3.64, *p *=* *.002, *d *=* *0.89. The rise in cortisol levels was significantly steeper in TD boys, *F*(1,70) = 5.39, *p *=* *.023, *η*_*p*_^2* *^= .072. The stress test also had an effect on the participants’ HR, in both boys with hfASD, *F*(2,92) = 85.99, *p *<* *.001, *η*_*p*_^2 ^= .651, and TD controls, *F*(1.35,25.66) = 74.30, *p *<* *.001, *η*_*p*_^2 ^= .903. TD boys again showed a stronger physiological reactivity to the stressor, *F*(1.65,108.91) = 8.58, *p *=* *.001, *η*_*p*_^*2 *^= .115. Overall, the PST was successful in producing self‐reported and physiological changes.

### Irritability and physiological responses to stress in hfASD

To explore the relation between irritability and stress‐induced changes in cortisol and HR in hfASD, we conducted repeated‐measures ANOVAs with irritability as a predictor. Age was added as a covariate in all cortisol analyses. For HR variability results please see Appendix S2.

#### Cortisol reactivity

**Figure 3:**
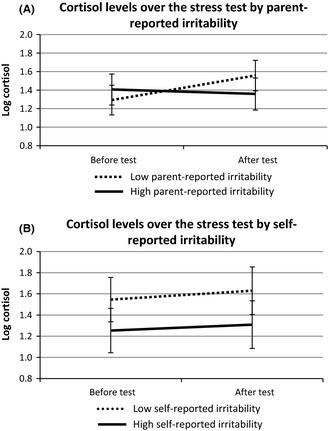
Pre and poststressor comparisons of cortisol levels for low versus high parent‐ and self‐reported irritability (median split) in boys with high‐functioning autism spectrum disorders (hfASD; 95% confidence intervals)

Parent‐report. We found a time‐by‐irritability interaction for parent‐report, *F*(1,39) = 8.71, *p *=* *.005, *η*_*p*_^2 ^= .182. Figure 3A illustrates this interaction schematically, using a median split to show that boys with high parent‐reported irritability had a relatively dampened cortisol response to stress compared to those with low parent‐reported irritability. We then examined whether anxiety affected the stress‐induced change in cortisol levels. However, the time‐by‐irritability interaction effect remained significant after adding parent‐reported anxiety into the model as a covariate, *F*(1,38) = 5.05, *p *=* *.031, *η*_*p*_^2 ^= .117. No independent significant effects of parent‐reported anxiety on cortisol levels were found when both irritability and anxiety were added into the model.

Self‐report. Irritability had a main effect on cortisol levels before and after the stressor, *F*(1,25) = 7.10, *p *=* *.013, *η*_*p*_^2 ^= .221. Compared to adolescents with low self‐reported irritability, those with high self‐reported irritability showed lower cortisol levels irrespective of time (Figure 3B). Similar to parent‐report, the effect of self‐reported irritability on cortisol levels remained significant after adding self‐reported anxiety to the model as a covariate, *F*(1,23) = 4.96, *p *=* *.036, *η*_*p*_^2 ^= .177. No significant effects of self‐reported anxiety on cortisol levels were found when both irritability and anxiety were added into the model.

#### Heart rate

**Figure 4:**
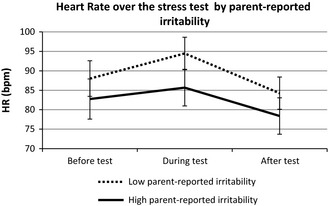
The relation between parent‐reported irritability (median split, low vs. high) and heart rate before, during and after the psychosocial stress test in boys with high‐functioning autism spectrum disorders (hfASD; 95% confidence intervals)

Parent‐report. We found a time‐by‐irritability interaction for parent‐report, *F*(1.65,64.24) = 6.10, *p *=* *.006, *η*_*p*_^2 ^= .135. Boys with high parent‐reported irritability displayed a dampened HR response to the stressor compared to boys with low parent‐reported irritability (Figure 4). However, this effect was no longer significant after parent‐reported anxiety was added into the model as a covariate. Instead, there was a significant interaction between parent‐reported anxiety and time, *F*(1.62,61.73) = 4.01, *p *=* *.031, *η*_*p*_^2 ^= .095. Boys with high parent‐reported anxiety displayed a dampened HR reaction to the stressor compared with boys with low parent‐reported anxiety.

Self‐report. No significant effects of self‐reported irritability or anxiety on HR reactivity to stress were found.

## Discussion

We showed that irritability can be measured reliably in boys with hfASD using a concise scale, and found a relation between irritability and physiological markers of stress response. Lower cortisol levels were observed in boys with high compared to low self‐reported irritability; and lower HR was noted in boys with high compared to low parent‐reported irritability. This was accompanied by a dampened physiological response to the experimental stressor in those with high parent‐reported irritability. The results were consistent across two statistical approaches used.

### Measurement of irritability in hfASD

Irritability showed high internal consistencies for both the parent‐ and self‐reported scales. Item frequency pattern for both parent‐ and self‐reported irritability in boys with hfASD mirrored that of boys with SMD. Consistent with previous results in TD children (Stringaris, Goodman, et al., 2012), we found a strong correlation between parent‐ and self‐reported irritability in boys with hfASD. Finally, both boys with hfASD and their parents found the impairment due to irritability to be directly proportional to the level of irritability symptoms. This is consistent with previous studies showing that children with ASD can be as good as their parents in reporting on certain aspects of their psychopathology (Knott, Dunlop, & Mackay, 2006; Ozsivadjian & Knott, 2011). Similarities between ARI scores in the SMD and hfASD groups suggest that irritability may be a critical dimension of the ASD phenotype. Additionally, the fact that irritability symptoms were impacting the lives of boys with hfASD argues for irritability to be a target for clinical interventions as in highly irritable TD children (Scott & O'Connor, 2012).

### Mechanisms of irritability in hfASD

We investigated whether irritability was associated with anxiety and stress‐response in boys with hfASD. Parent, but not self‐reports of irritability and anxiety were positively correlated, which may be due to introspection difficulties or alexithymia, a condition characterized by difficulties in identifying and describing emotions (e.g., Silani et al., 2008). Future studies should examine this possibility, since introspection difficulties and alexithymia were not measured in our study. However, this seems unlikely based on similar parent‐ and self‐reporting on irritability in our hfASD group and the fact that boys with hfASD found the PST as stressful as TD boys did.

### Cortisol findings

The stress‐induced increase in cortisol levels in hfASD was smaller than the increase in TD boys, despite an equally strong rise in subjective stress‐response across both groups. Boys with hfASD reporting high levels of irritability had significantly lower levels of cortisol; also boys who were rated as highly irritable by their parents showed blunted cortisol reactivity to stress. Low overall cortisol levels and dampened cortisol reactivity to psychosocial stress are often reported in children with ASD (Lanni et al., 2012; Levine et al., 2012), although the literature is inconsistent (Jansen et al., 2003) and cortisol levels in hfASD are within the normal range. Interestingly, lower cortisol levels throughout a psychosocial stress test have also been reported in TD boys with ODD (van Goozen et al., 1998) – a disorder characterized by irritability (Krieger et al., 2013; Stringaris & Goodman, 2009b). In addition, low plasma levels of cortisol also feature in posttraumatic stress disorder (PTSD; Yehuda, 2001), a disorder where irritability and anxiety are prominent. We found no independent contribution of anxiety to cortisol stress responses, consistent with a previous psychosocial stress study in boys with hfASD (Simon & Corbett, 2013). One possibility is that highly irritable adolescents with hfASD are particularly liable to experience chronic stress. This is consistent with studies that reported decreased cortisol responses to stress in long‐lasting psychopathology, including PTSD (Yehuda, 2001; Yehuda & Seckl, 2011) and chronic adolescent depression (Booij, Bouma, de Jonge, Ormel, & Oldehinkel, 2013).

### Heart rate findings

Although for parent‐reported irritability the pattern of HR results was similar to our findings with cortisol, the effects became nonsignificant after adding parent‐reported anxiety into the model. Instead, boys with high parent‐reported anxiety displayed dampened HR reaction to stress compared with boys with low parent‐reported anxiety. This pattern of HR reactivity may reflect stress‐induced physiological withdrawal of boys with hfASD who were rated as highly anxious, based on our previous findings where children with ASD and anxiety showed reduced HR responsiveness to stress that was significantly related to anxiety severity (Hollocks, Howlin, Papadopoulos, Khondoker, & Simonoff, 2014). An alternative view could be that cortisol and HR responses to stress are qualitatively different, since HPA‐axis reactivity has a slower onset than the sympathetic system (Bauer, Quas, & Boyce, 2002). However, our HRV analyses revealed no relation between irritability and sympathetic system activity. Instead, irritability was related to parasympathetic activity, although its effect lost significance after adding anxiety into the model.

### Strengths and limitations

The strengths of this study include an experimental design and the use of multiple physiological measures: cortisol, HR, and HRV. All participants were medication‐free, ensuring that the differences in physiology were unconfounded by treatment status. However, it is unclear whether the findings generalize to those individuals with ASD who take psychotropic medications. The study is limited by its modest sample size and unequal sample sizes between the groups. Due to the lack of variance in irritability scores among TD participants who took part in the PST, we were unable to compare the effects of irritability on physiological stress‐responsiveness across boys with and without hfASD. A future study is needed for this purpose. Motor movement controls were not thoroughly assessed in the HR analysis, although it is noteworthy that using the BioHarness usually results in less movement artefacts compared to wire‐based systems. ASD diagnoses could not be confirmed using structured assessments in all cases, although care was taken to limit the chance of false positives. Our study did not assess the full range of comorbidities such as depression (closely linked to irritability). Finally, future studies on HR responsiveness would benefit from employing time‐series analyses such as autoregressive integrated moving‐average (ARIMA) models to account for serial dependency in individual HR data (Goodwin et al., 2006; Groden et al., 2005).

## Conclusion

Our results suggest that irritability can be reliably measured by both parent‐ and self‐report in boys with hfASD and that it may shape physiological responses to stress in this population. This may have important clinical implications, as dampened cortisol responsiveness to stress predicted poorer treatment outcomes in TD children with disruptive behavior disorder (van de Wiel, van Goozen, Matthys, Snoek, & van Engeland, 2004). It therefore seems important to investigate the mechanisms of physiological underactivation in ASD during stress and clarify the role of irritability and anxiety in the process.

**Key points**

- Irritability is common in children with ASD but has been understudied.
- Irritability can be reliably measured in youth with hfASD.
- Clinicians may benefit from using both parent‐ and child‐rated scales to get a comprehensive view of the child's irritability.
- Children with hfASD who are highly irritable showed lower cortisol levels, lower heart rate and a blunted physiological response to stress.
- Dampened stress reactivity may be an important pathophysiological mechanism in children with hfASD and irritability.

## Supporting information


**Appendix S1.** Additional references to studies that investigated heart rate in youth with ASD.
**Appendix S2.** Heart rate variability analyses in boys with hfASD and TD controls.
**Appendix S3.** Piecewise regression models examining physiological responses to stress in boys with hfASD.
