# Supplementary material for: Irritability in boys with autism spectrum disorders: an investigation of physiological reactivity
Source: J Child Psychol Psychiatry. 2015 Jan 28;56(10):1118–26. doi: 10.1111/jcpp.12382 (PMC4737220; doi:10.1111/jcpp.12382)
Supplement: Supplementary file 1 — Appendix S1. Additional references to studies that investigated heart rate in youth with ASD. Appendix S2. Heart rate variability analyses in boys with hfASD and TD controls. Appendix S3. Piecewise regression models examining physiological responses to stress in boys with hfASD. [file JCPP-56-1118-s001.docx]

***Online supplementary material for: Irritability in boys with autism spectrum disorders: an investigation of physiological reactivity; by Mikita et al***

[**Appendix S1. Additional references to studies that investigated heart rate in children and adolescents with ASD.**](#S1)

[**Appendix S2. Heart rate variability analyses in boys with high-functioning autism spectrum disorders (hfASD) and typically-developing (TD) controls.**](#S2)

[**Appendix S3. Piecewise regression models examining physiological responses to psychosocial stress in boys with high-functioning autism spectrum disorders (hfASD).**](#S3)

**Appendix S1. Additional references to studies that investigated heart rate (HR) in children and adolescents with ASD.**

1. HR responsiveness to stress in youth with ASD

Corona, R., Dissanayake, C., Arbelle, S., Wellington, P., & Sigman, M. (1998). Is affect aversive to young children with autism? Behavioral and cardiac responses to experimenter distress. *Child Dev, 69*(6), 1494-1502.

Groden, J., Goodwin, M. S., Baron, M. G., Groden, G., Velicer, W. F., Lipsitt, L. P., . . . Plummer, B. (2005). Assessing Cardiovascular Responses to Stressors in Individuals With Autism Spectrum Disorders. *Focus on Autism and Other Developmental Disabilities, 20*(4), 244-252.

Kushki, A., Drumm, E., Pla Mobarak, M., Tanel, N., Dupuis, A., Chau, T., & Anagnostou, E. (2013). Investigating the autonomic nervous system response to anxiety in children with autism spectrum disorders. *PLoS One, 8*(4), e59730. doi: 10.1371/journal.pone.0059730

Sheinkopf, S. J., Neal-Beevers, A. R., Levine, T. P., Miller-Loncar, C., & Lester, B. (2013). Parasympathetic response profiles related to social functioning in young children with autistic disorder. *Autism Res Treat, 2013*, 868396. doi: 10.1155/2013/868396

Sigman, M., Dissanayake, C., Corona, R., & Espinosa, M. (2003). Social and cardiac responses of young children with autism. *Autism, 7*(2), 205-216.

Willemsen-Swinkels, S. H., Bakermans-Kranenburg, M. J., Buitelaar, J. K., van, I. M. H., & van Engeland, H. (2000). Insecure and disorganised attachment in children with a pervasive developmental disorder: relationship with social interaction and heart rate. *J Child Psychol Psychiatry, 41*(6), 759-767.

1. HR responsiveness to other tasks and stimuli in youth with ASD

Althaus, M., Mulder, L. J., Mulder, G., Aarnoudse, C. C., & Minderaa, R. B. (1999). Cardiac adaptivity to attention-demanding tasks in children with a pervasive developmental disorder not otherwise specified (PDD-NOS). *Biol Psychiatry, 46*(6), 799-809.

Bernal, M. E., & Miller, W. H. (1970). Electrodermal and cardiac responses of schizophrenic children to sensory stimuli. *Psychophysiology, 7*(2), 155-168.

Daluwatte, C., Miles, J. H., Christ, S. E., Beversdorf, D. Q., Takahashi, T. N., & Yao, G. (2013). Atypical pupillary light reflex and heart rate variability in children with autism spectrum disorder. *J Autism Dev Disord, 43*(8), 1910-1925. doi: 10.1007/s10803-012-1741-3

MacCulloch, M. J., & Williams, C. (1971). On the nature of infantile autism. *Acta Psychiatr Scand, 47*(3), 295-314.

Porges, S. W., Macellaio, M., Stanfill, S. D., McCue, K., Lewis, G. F., Harden, E. R., . . . Heilman, K. J. (2013). Respiratory sinus arrhythmia and auditory processing in autism: modifiable deficits of an integrated social engagement system? *Int J Psychophysiol, 88*(3), 261-270. doi: 10.1016/j.ijpsycho.2012.11.009

Watson, L. R., Roberts, J. E., Baranek, G. T., Mandulak, K. C., & Dalton, J. C. (2012). Behavioral and physiological responses to child-directed speech of children with autism spectrum disorders or typical development. *J Autism Dev Disord, 42*(8), 1616-1629. doi: 10.1007/s10803-011-1401-z

**Appendix S2. Heart rate variability analyses in boys with high-functioning autism spectrum disorders (hfASD) and typically-developing (TD) controls.**

**Methods**

Heart Rate (HR) was recorded continuously throughout the psychosocial stress test (PST). HR electrocardiogram (ECG) was measured at 250_Hz_ using the Zephyr BioHarness wireless telemetry system. The BioHarness is a small, lightweight device worn around the chest via an unobtrusive strap that records human physiology signals, including ECG, respiration rate and temperature. It was shown to be reliable and valid (Johnstone, Ford, Hughes, Watson, & Garrett, 2012a, 2012b) in laboratory and field settings (Johnstone, Ford, Hughes, Watson, Mitchell, et al., 2012). The ECG signal was analyzed with Labchart 7 HRV module (ADInstruments Pty Ltd, Bella Vista, Australia). The ECG recording was segmented into nine 5-minute long segments selected across the 100 minutes of recording, as recommended by the Task Force (1996). Three blocks were taken from the rest phase, two from speech preparation, one from speech and three from the recovery phase. The signal was pre-processed with a low-pass filter before R-wave to R-wave (RR) intervals were automatically identified. Detected RR intervals were then manually inspected for errors. Ectopic RR intervals were excluded and subsequently interpolated and replaced by the average of nearest preceding and succeeding normal – normal intervals (NN) within the block. We conducted a spectral HRV analysis using Fast Fourier Transform. HRV falling within the high-frequency domain (HF; .15-.40Hz) is considered a measure of respiratory sinus arrhythmia and lower values indicate lower parasympathetic modulation. To isolate the relative contribution of the sympathetic modulation, we calculated a ratio between the low-frequency domain (LF; .04-.15Hz) and HF (methodology further described in Hollocks, Howlin, Papadopoulos, Khondoker, & Simonoff, 2014).

**Results**

HR variability changed significantly throughout the PST in boys with hfASD [spectral analysis: *F*(2,78)=51.42, *p*<.001, *η_p_*^2^=.569; LF/HF ratio analysis: *F*(1.44,56.17)=4.44, *p*=.027, *η_p_*^2^=.102]. TD boys displayed a change in HR variability in the spectral [*F*(1,19)=33.58, *p*<.001, *η_p_*^2^=.636] but not ratio analysis [*F*(2,38), *p*=.419].

(See also Table S2 for individual mean cortisol and heart rate results for all participants)

**Table S1.** Means (standard deviations, ranges) and sample sizes for heart rate variability by hfASD and TD control groups.

|  | **hfASD** | **n** | **TD controls** | **n** |
| --- | --- | --- | --- | --- |
|  |  |  |  |  |
| **Psychosocial Stress Test** | |  |  |  |
|  |  |  |  |  |
| Heart rate variability: spectral analysis (NU) | |  |  |  |
| before test | 53.4 (17.0, 24.1-96.3) | 46 | 50.6 (15.7, 32.4-88.3) | 21 |
| during test | 32.7 (12.9, 10.4-60.0) | 49 | 30.6 (9.8, 12.6-48.3) | 20 |
| after test | 53.8 (14.9, 26.2-83.9) | 45 | 47.7 (16.9, 21.3-77.1) | 20 |
| Heart rate variability: LF/HF ratio analysis | |  |  |  |
| before test | 1.7 (1.0, 0.5-4.3) | 46 | 1.9 (0.9, 0.6-3.3) | 21 |
| during test | 2.2 (1.7, 0.4-8.1) | 49 | 2.3 (1.4, 0.8-6.0) | 20 |
| after test | 1.7 (1.0, 0.3-4.1) | 45 | 2.3 (1.4, 0.6-5.3) | 20 |

LF/HF low frequency/high frequency.

*Heart rate variability: High-frequency spectral analysis.*

Parent-report. We found a main effect of irritability, *F*(1,32)=4.68, *p*=.038, *η_p_*^2^=.127. Boys with high parent-reported irritability displayed overall higher parasympathetic modulation compared to those with low parent-reported irritability (Figure S1). However, this effect was no longer significant after parent-reported anxiety was added into the model as a covariate. Instead, there was a main effect of parent-reported anxiety, *F*(1,31)=4.50, *p*=.042, *η_p_*^2^=.127. Boys with high parent-reported anxiety displayed higher parasympathetic modulation compared to boys with low parent-reported anxiety.

Self-report. No significant effects of self-reported irritability or anxiety on parasympathetic activity were found.

**Figure S1.** The relation between parent-reported irritability (median split, low vs. high) and parasympathetic activity before, during and after the psychosocial stress test in boys with high-functioning autism spectrum disorders (hfASD) (95% confidence intervals).

*Heart rate variability: LF/HF ratio analysis.*

Parent-report. We found no effects of parent-reported irritability on sympathetic activity. There was an independent main effect of parent-reported anxiety [*F*(1,37)=4.74, *p*=.036, *η_p_*^2^=.114], with boys rated as highly-anxious by their parents displaying lower sympathetic activity.

Self-report. No significant effects of self-reported irritability or anxiety on sympathetic activity were found.

**Table S2. Individual mean cortisol and heart rate results for all participants.**

|  | **Cortisol (log)** | | **Heart Rate (bpm)** | | |
| --- | --- | --- | --- | --- | --- |
|  | before test | after test | before test | during test | after test |
|  |  |  |  |  |  |
| **Boys with hfASD** | |  |  |  |  |
|  |  |  |  |  |  |
| 1 | 1.5 | 1.4 | 67.9 | 72.9 | 63.5 |
| 2 | 1.7 | 1.7 | 96.0 | 104.6 | 105.3 |
| 3 | 1.6 | 1.7 | 83.9 | 89.5 | 84.1 |
| 4 | 1.3 | 1.4 | 81.7 | 86.9 | 76.1 |
| 5 | 2.1 | 1.6 | 85.9 | 79.4 | 72.9 |
| 6 | 1.1 | 1.3 | 92.8 | 85.4 | 82.9 |
| 7 | 1.0 | 1.1 | 91.3 | 89.9 | 92.5 |
| 8 | 1.2 | 1.0 | 83.2 | 82.2 | 77.6 |
| 9 | 1.1 | 1.6 | 62.9 | 67.1 | 57.4 |
| 10 | 1.2 | 1.3 | 75.3 | 81.3 | 76.5 |
| 11 | 1.7 | n/a | 103.1 | 106.5 | 95.5 |
| 12 | 1.5 | 2.0 | 92.1 | 99.1 | 93.4 |
| 13 | 0.7 | 1.0 | 77.4 | 77.5 | 74.5 |
| 14 | 1.2 | n/a | 77.4 | 84.1 | n/a |
| 15 | 1.5 | 1.4 | 84.8 | 87.3 | 78.3 |
| 16 | 0.9 | 1.5 | 73.7 | 75.5 | 67.9 |
| 17 | 1.5 | 1.3 | 78.9 | 83.0 | 76.3 |
| 18 | 1.3 | 0.9 | 83.0 | 88.3 | 75.5 |
| 19 | 0.6 | 0.5 | 85.0 | 86.9 | 79.3 |
| 20 | 0.8 | 1.3 | 82.5 | 94.0 | 84.5 |
| 21 | 1.7 | 1.6 | 94.0 | 95.3 | 90.8 |
| 22 | 1.0 | 1.2 | 102.1 | 109.0 | 98.3 |
| 23 | 1.6 | 1.6 | 83.7 | 95.2 | 80.5 |
| 24 | 2.1 | 1.9 | n/a | n/a | n/a |
| 25 | 1.0 | 1.8 | 84.9 | 84.8 | 81.1 |
| 26 | 1.6 | 1.7 | 108.9 | 120.8 | 104.1 |
| 27 | 1.3 | 1.2 | 91.9 | 96.8 | 87.6 |
| 28 | 1.1 | 2.0 | 94.9 | 97.7 | 89.2 |
| 29 | 2.2 | 2.2 | 86.4 | 100.8 | 83.2 |
| 30 | 1.0 | 1.0 | 74.6 | 78.0 | 73.2 |
| 31 | 1.1 | 0.8 | 84.1 | 84.6 | 79.5 |
| 32 | 1.0 | 1.4 | 70.8 | 87.2 | 69.3 |
| 33 | 1.6 | 1.1 | 77.8 | 84.0 | 71.4 |
| 34 | 1.5 | 1.9 | 74.6 | 86.8 | 70.9 |
| 35 | 1.6 | 1.6 | 65.6 | 67.6 | 62.1 |
| 36 | 1.0 | 0.6 | 75.7 | 86.0 | 76.8 |
| 37 | 1.3 | 2.0 | 75.8 | 81.7 | 71.2 |
| 38 | 1.2 | 1.3 | 68.5 | 73.2 | 64.4 |
| 39 | 2.0 | 2.0 | 78.0 | 94.6 | 79.0 |
| 40 | 0.9 | 0.8 | 86.3 | 87.9 | 82.3 |
| 41 | 0.8 | 2.0 | 85.9 | 90.5 | 80.6 |
| 42 | 2.4 | 1.8 | 92.4 | 89.4 | 85.9 |
| 43 | 1.0 | 1.2 | 85.5 | 92.1 | 83.7 |
| 44 | 1.3 | 1.7 | 85.3 | 96.8 | 84.5 |
| 45 | 1.6 | 0.9 | 102.5 | 103.5 | 95.5 |
| 46 | 1.3 | 2.2 | 70.2 | 83.5 | 68.8 |
| 47 | 1.3 | 1.6 | 92.3 | 92.9 | 91.0 |
| 48 | 1.0 | 1.2 | 109.8 | 113.0 | 97.4 |
| 49 | 1.2 | 2.0 | 107.7 | 108.2 | 102.6 |
| 50 | 1.7 | 1.5 | 75.6 | 80.7 | 67.7 |
| 51 | 1.5 | 1.6 | 84.9 | n/a | 78.9 |
| 52 | 1.8 | 1.5 | 77.0 | 80.7 | n/a |
|  |  |  |  |  |  |
| **TD boys** | |  |  |  |  |
|  |  |  |  |  |  |
| 53 | 1.5 | 1.7 | 84.8 | 94.0 | 81.1 |
| 54 | 1.6 | 1.6 | 67.1 | 74.3 | 70.3 |
| 55 | 1.4 | 1.3 | 60.2 | 76.4 | 59.1 |
| 56 | 1.4 | 1.4 | 85.5 | 103.9 | n/a |
| 57 | 1.3 | 2.6 | 74.8 | 80.5 | 66.4 |
| 58 | 0.9 | 1.3 | 70.7 | 89.3 | 72.0 |
| 59 | 2.0 | 2.1 | 72.4 | 95.9 | 73.3 |
| 60 | 1.0 | 2.1 | 61.3 | 72.2 | 60.6 |
| 61 | 1.6 | 1.8 | 88.9 | 90.6 | 84.7 |
| 62 | 0.7 | 1.3 | 65.1 | 66.6 | 59.5 |
| 63 | 1.6 | 1.8 | 81.2 | 88.6 | 79.5 |
| 64 | 1.1 | 1.0 | 68.2 | 69.1 | 60.6 |
| 65 | 1.2 | 1.8 | 85.4 | 91.2 | 78.9 |
| 66 | 1.8 | 1.9 | 75.0 | 83.9 | 75.8 |
| 67 | 1.7 | 1.7 | 74.9 | 84.1 | 73.3 |
| 68 | 1.1 | 2.2 | 82.3 | 93.2 | 78.6 |
| 69 | 1.7 | 2.1 | 77.4 | n/a | n/a |
| 70 | 1.8 | 2.0 | 74.4 | 83.9 | 70.9 |
| 71 | 1.6 | 3.1 | 83.1 | 103.5 | 83.9 |
| 72 | 1.5 | 1.8 | 93.6 | 101.8 | 90.5 |
| 73 | 1.3 | n/a | 89.8 | 94.4 | n/a |
| 74 | 1.4 | 1.1 | 75.0 | 89.0 | 74.8 |
| 75 | 0.8 | 1.4 | 68.7 | 76.1 | 64.8 |

**References**

Hollocks, M. J., Howlin, P., Papadopoulos, A. S., Khondoker, M., & Simonoff, E. (2014). Differences in HPA-axis and heart rate responsiveness to psychosocial stress in children with autism spectrum disorders with and without co-morbid anxiety. *Psychoneuroendocrinology, 46*(0), 32-45. doi: <http://dx.doi.org/10.1016/j.psyneuen.2014.04.004>

Johnstone, J. A., Ford, P. A., Hughes, G., Watson, T., & Garrett, A. T. (2012a). Bioharness(TM) multivariable monitoring device: part. I: validity. *J Sports Sci Med, 11*(3), 400-408.

Johnstone, J. A., Ford, P. A., Hughes, G., Watson, T., & Garrett, A. T. (2012b). Bioharness(TM) Multivariable Monitoring Device: Part. II: Reliability. *J Sports Sci Med, 11*(3), 409-417.

Johnstone, J. A., Ford, P. A., Hughes, G., Watson, T., Mitchell, A. C., & Garrett, A. T. (2012). Field based reliability and validity of the bioharness multivariable monitoring device. *J Sports Sci Med, 11*(4), 643-652.

Task Force of the European Society of Cardiology and the North American Society of Pacing

and Electrophysiology. (1996). Heart rate variability. Standards of measurement,

physiological interpretation, and clinical use. *Eur Heart J, 17*(3), 354-381.

**Appendix S3. Piecewise regression models examining physiological responses to psychosocial stress in boys with high-functioning autism spectrum disorders (hfASD).**

The response profiles of cortisol and HR (average values at all time points and slope decline/increase rates) exhibited non-linear relationships with time. Piecewise models can fit any type of non-linear relationships by assuming parametric relationships within smaller segments (Marsh & Cormier, 2001). Unlike ANOVAs, they allow formal hypothesis-testing about the mean and slope of the response profile within segments. We fitted piecewise linear mixed models to the non-linear profiles by dividing the time axis (all time points) into three segments using pre-specified knot points. The results complemented our ANOVAs and provided a stringent test of differences between physiological response profiles of adolescents with hfASD who were low vs. high on irritability.

***Method***

Based on an exploratory analysis of the response profiles, we fitted three-piece linear mixed models using two knot points, one at just before the initiation of the psychosocial stress and the other corresponding to the peak/nadir of the stress response profile. For cortisol, the peak of the stress response profile corresponded to the saliva sample obtained post-stressor (+20min). For HR, the peak of the stress response profile corresponded to HR readings averaged over the 20-min stress phase. This partitioning allows convenient modelling of the physiological parameter profiles by using different parameterization for the rest, stress and recovery periods and allowing hypothesis-testing to compare the parameters within periods.

The analyses were performed separately for self- and parent-reported irritability. Participants were assigned into groups of low vs. high irritability using a median split on the total irritability score. We then tested the role of anxiety in shaping physiological stress response profiles, by fitting a separate piecewise regression model with anxiety (total SCAS score, self- or parent-reported) into the model as a covariate.

***Cortisol reactivity***

A piecewise regression model was fitted to *log* cortisol data across each of the rest, stress and recovery phases of the psychosocial stress test. Importantly, as can be seen in Table S3, there were no significant group differences in either the rest or the recovery phase slopes, for both self- and parent-reported irritability.

*Parent-report.* In the transition from rest to stress, those rated as highly irritable by their parents had a significantly blunted cortisol response slope compared to those low on irritability, even after adding parent-reported anxiety into the model as a covariate (*β* = -0.27, *p* = .001). These results mirror the significant time by irritability interaction obtained using a repeated measures ANOVA reported in the main text.

*Self-report.* There were significant mean differences between boys who rated themselves as high vs. low on irritability, both at the first knot point (pre-stress; *β_0_* = -0.29, *p* = .04) and at the second knot point (post-stress, *β_0_* = -0.30, *p* = .04), with those high on irritability showing lower mean cortisol values. This is consistent with the main effect of self-reported irritability reported in the main text. However, these mean differences were marginally no longer significant after adding anxiety into the model as a covariate.

***Heart rate***

Another piecewise regression model was fitted to the time-series mean HR data across the rest, stress and recovery phases of the psychosocial stress test. Similarly to cortisol findings, we did not find significant group differences in either the rest or the recovery phase slopes, for both self- and parent-reported irritability (see Table S3).

*Parent-report.* In the transition from rest to stress, those rated as highly irritable by their parents had a significantly blunted HR response slope compared to those low on irritability (*β* = -3.27, *p* = .03). The results remained significant after adding parent-reported anxiety into the model as a covariate (*β* = -3.27, *p* = .03). Boys who scored high on irritability also displayed lower mean HR at the second knot point (post-stress) compared to those low on irritability (*β_0_* = -7.98, *p* = .01). This effect remained significant after adding self-reported anxiety into the model as a covariate (*β_0_* = -7.65, *p* = .03).

*Self-report.* No significant intercept or slope differences were found.

***References***

Marsh, L. C., & Cormier, D. R. (2001). *Spline regression models*: Sage.

Table S3. Piecewise regression model statistics for cortisol and heart rate responses to psychosocial stress in boys with high-functioning ASD.

|  |  | **Rest slope difference** | | | | **Mean difference at Pre-stress (Knot point: just before stressor)** | | | | **Stress Slope Difference** | | | | **Mean difference Post stress**  **(Knot Point: peak stress response)** | | | | **Recovery Slope Difference** | | | |
| --- | --- | --- | --- | --- | --- | --- | --- | --- | --- | --- | --- | --- | --- | --- | --- | --- | --- | --- | --- | --- | --- |
|  | **Cortisol** | Coef. | SE | *p* | 95% CI | Coef. | SE | *p* | 95% CI | Coef. | SE | *p* | 95% CI | Coef. | SE | *p* | 95% CI | Coef. | SE | *p* | 95% CI |
| parent report | high vs. low irritability | 0.06 | 0.05 | .29 | -.05 - .16 | 0.08 | 0.12 | .48 | -.15 - .31 | -0.27 | 0.08 | **.001** | -.43 - -.11 | -0.19 | 0.12 | .12 | -.43 - .05 | 0.09 | 0.05 | .10 | -.02 - .19 |
|  | … covarying for anxiety | 0.06 | 0.05 | .29 | -.05 - .16 | 0.07 | 0.13 | .57 | -.18 - .32 | -0.27 | 0.08 | **.001** | -.43 - -.11 | -0.20 | 0.13 | .12 | -.46 - .06 | 0.09 | 0.05 | .10 | -.02 - .19 |
| self report | high vs. low irritability | -0.06 | 0.07 | .38 | -.21 - .08 | -0.29 | 0.14 | **.04** | -.57 - -.009 | -0.02 | 0.12 | .89 | -.24 - .21 | -0.30 | 0.15 | **.04** | -.60 - -.008 | 0.03 | 0.07 | .68 | -.11 - .18 |
|  | … covarying for anxiety | -0.06 | 0.07 | .40 | -.21 - .08 | -0.29 | 0.15 | .05 | -.58 - .001 | 0.02 | 0.12 | .89 | -.22 - .25 | -0.27 | 0.16 | .08 | -.58 - .04 | 0.02 | 0.08 | .78 | -.13 - .17 |
|  |  |  |  |  |  |  |  |  |  |  |  |  |  |  |  |  |  |  |  |  |  |
|  | **Heart Rate** |  |  |  |  |  |  |  |  |  |  |  |  |  |  |  |  |  |  |  |  |
| parent report | high vs. low irritability | 1.65 | 1.54 | .28 | -1.37 - 4.67 | -4.71 | 3.23 | .15 | -11.03 - 1.62 | -3.27 | 1.49 | **.03** | -6.19 - -.36 | -7.98 | 3.21 | **.01** | -14.26 - -1.70 | 1.57 | 0.81 | .05 | -.006 - 3.15 |
|  | … covarying for anxiety | 1.65 | 1.54 | .28 | -1.37 - 4.67 | -4.38 | 3.48 | .21 | -11.21 - 2.44 | -3.27 | 1.49 | **.03** | -6.18 - -.36 | -7.65 | 3.46 | **.03** | -14.44 - -.87 | 1.57 | 0.81 | .05 | -.006 - 3.15 |
| self report | high vs. low irritability | -0.23 | 1.78 | .90 | -3.72 - 3.26 | 0.17 | 3.92 | .96 | -7.50 - 7.85 | -1.17 | 1.72 | .50 | -4.55 - 2.21 | -1.00 | 3.89 | .80 | -8.62 - 6.63 | -0.96 | 0.94 | .31 | -2.81 - .89 |
|  | … covarying for anxiety | -0.35 | 1.84 | .85 | -3.96 - 3.26 | 0.74 | 4.07 | .86 | -7.23 - 8.72 | -0.95 | 1.78 | .60 | -4.44 - 2.55 | -0.20 | 4.04 | .96 | -8.13 - 7.73 | -0.95 | 0.98 | .33 | -2.87 - .96 |
|  |  |  |  |  |  |  |  |  |  |  |  |  |  |  |  |  |  |  |  |  |  |

SE, standard error; CI, confidence interval. *Rest slope* encompassed all time points before the stressor. *Stress slope* consisted of two time points: just before stressor and peak stress response. *Recovery Slope* started at peak stress response and encompassed all remaining time points.
